# Aging, Cellular Senescence, and Alzheimer’s Disease

**DOI:** 10.3390/ijms23041989

**Published:** 2022-02-11

**Authors:** Rui-Ming Liu

**Affiliations:** Division of Pulmonary, Allergy, and Critical Care, Department of Medicine, School of Medicine, University of Alabama at Birmingham, Birmingham, AL 35294-0006, USA; rliu@uab.edu; Tel.: +1-(205)-934-7028; Fax: +1-(205)-934-1721

**Keywords:** aging, cellular senescence, Alzheimer’s disease, neurodegeneration, late-onset Alzheimer’s disease (LOAD), telomere shortening, β-amyloid peptides (Aβ), tauopathy, oxidative stress, plasminogen activator inhibitor 1 (PAI-1)

## Abstract

Aging is the greatest risk factor for late-onset Alzheimer’s disease (LOAD), which accounts for >95% of Alzheimer’s disease (AD) cases. The mechanism underlying the aging-related susceptibility to LOAD is unknown. Cellular senescence, a state of permanent cell growth arrest, is believed to contribute importantly to aging and aging-related diseases, including AD. Senescent astrocytes, microglia, endothelial cells, and neurons have been detected in the brain of AD patients and AD animal models. Removing senescent cells genetically or pharmacologically ameliorates β-amyloid (Aβ) peptide and tau-protein-induced neuropathologies, and improves memory in AD model mice, suggesting a pivotal role of cellular senescence in AD pathophysiology. Nonetheless, although accumulated evidence supports the role of cellular senescence in aging and AD, the mechanisms that promote cell senescence and how senescent cells contribute to AD neuropathophysiology remain largely unknown. This review summarizes recent advances in this field. We believe that the removal of senescent cells represents a promising approach toward the effective treatment of aging-related diseases, such as AD.

## 1. Introduction

**Figure 1:**
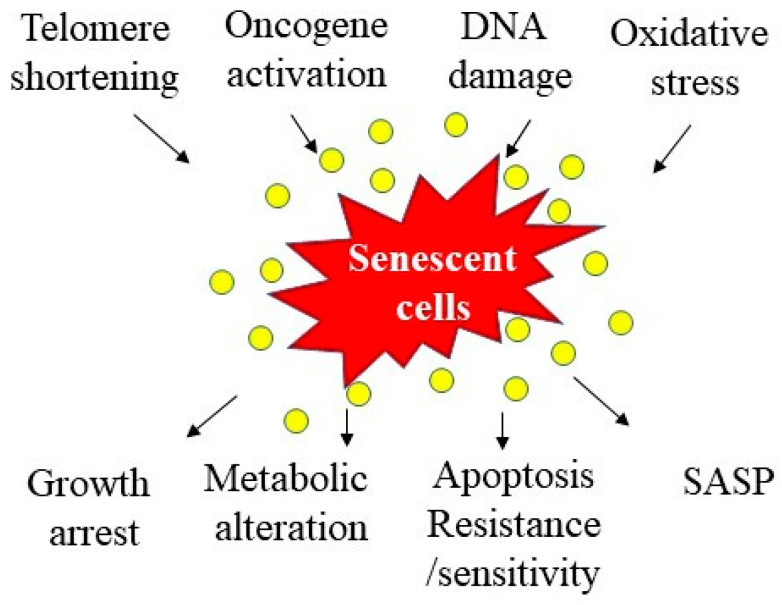
Potential causes and consequences of cell senescence. Several factors have been identified to induce cell senescence, including telomere shortening, oncogene activation, DNA damage, and oxidative stress. Senescent cells, on the other hand, exhibit multiple characteristics, including growth arrest, metabolic changes, altered apoptosis sensitivity, and senescence-associated secretary phenotype (SASP).

Cellular senescence, including replicative senescence (RS) and stress-induced premature senescence (SIPS), represents a state of permanent cell growth arrest [1,2,3]. Cellular senescence can occur at any life stage from embryo to adulthood, although it is associated with the aging process [4,5,6]. Cellular senescence is a highly regulated process and has both beneficial and detrimental effects, involved in embryonic development, wound healing, tumor suppression, and aging [4,5,6]. Cancer cells proliferate indefinitely; cancer cell senescence, therefore, is believed to be beneficial as it prevents the development of cancer. In many other cases, cell senescence is considered deleterious, as it recaptures aging and contributes to aging-related diseases [7,8,9,10,11,12,13,14,15]. Senescent cells display three major characteristics—loss of proliferation or regeneration capacity, alteration of metabolic functions and resistance to apoptosis, and secretion of an array of pathogenically active molecules, termed senescence-associated secretary phenotype (SASP) [1,16] (Figure 1).

The markers used to identify senescent cells include increased activity of senescence-associated β-galactosidase (SA-β-gal), revealed by x-gal staining, increased expression of cyclin-dependent kinase inhibitors/cell cycle repressors p16, p53, and p21, decreased phosphorylation of retinoblastoma protein (pRb), and increased cytological markers such as senescence-associated heterochromatin foci (SAHFs) and senescence-associated DNA damage foci (SDFs) [1,16,17]. Morphologically, senescent cells are enlarged and flattened [1,16,17]. In the culture, the best way to reveal senescent cells is to show cell cycle arrest using flow cytometry techniques. Increased expression of cell cycle repressors and SA-β-gal activity, as well as senescent morphological changes, can be easily revealed and correlated with cell cycle repression in replicative senescence (RS) or stress-induced premature senescence (SIPS) cells in culture. However, it is still a challenge to detect or reveal senescent cells in the tissues/organs in vivo, due to nonspecificity and low sensitivity of currently used senescence markers. It is recommended that multiple markers be used to reveal senescent cells, especially in vivo.

Although cellular senescence has been demonstrated to contribute importantly to aging and aging-related diseases [8,13,14,18,19], the causes of cellular senescence under either physiological or pathological conditions remain unclear. Several hypotheses, including telomere shortening, oncogene activation, DNA damage, chromatin perturbation, and stress, have been proposed [1] (Figure 1). It should be stressed that different factors may contribute to cell senescence under different pathological conditions or for different types of cells. The potential mechanisms underlying brain cell senescence in AD will be discussed later in this review article. Aside from the causes of cell senescence, it is also largely unknown how senescent cells promote aging and aging-related diseases. Progenitor cells or stem cells are particularly sensitive to senescence stresses. Therefore, one of the hypotheses is that cellular senescence leads to exhaustion of progenitor cells or stem cells, which, in turn, causes a decline in tissue regenerative capacity during aging or upon injury. Another potential mechanism whereby senescent cells contribute to aging and aging-related diseases is through secretion of pathologically active molecules, termed as senescence-associated secretary phenotype (SASP). These biologically active molecules can exert profound effects on secreting cells (autocrine function) or on neighboring cells (paracrine functions). Nonetheless, despite an incomplete understanding of the molecular mechanisms that promote cell senescence and by which senescent cells contribute to aging and aging-related diseases, animal studies and clinical trials have shown some promising results with senolytic drugs. Several recent studies have shown that removal of senescent cells pharmacologically or genetically slows down aging processes and alleviates the aging-related pathophysiological changes in several aging-related disease models [11,20,21,22,23,24,25,26]. Further elucidation of the mechanisms underlying cell senescence and whereby senescent cells contribute to the pathogenesis of diseases may lead to the development of novel therapeutics for the treatment of these aging-related diseases.

## 2. Cellular Senescence and Aging

The cause of aging, an inevitable biological process that affects almost all living organisms, is still an area of significant controversy. Although numerous hypotheses, including increased oxidative stress, accumulation of damaged DNA molecules/mutations, and telomere shortening [27,28], have been proposed, none of these hypotheses completely explains aging phenotypes or process. It has long been appreciated that cellular senescence is associated with aging and may underlie the pathophysiology associated with aging. Nonetheless, although it has long been speculated that cell senescence underlies the pathophysiology of aging and aging-related diseases, there is no direct evidence to support the causative relation between cellular senescence and aging-related decline in organ function or diseases in vivo, until recent years, when p16 deficient mice and senolytic drugs became available [8]. These novel genetic and pharmacological tools help elucidate the significant contribution of cellular senescence to aging and aging-related diseases.

The relation between cell senescence and aging was mainly supported by correlation studies at the beginning. Increased expression of the heterochromatin-associated protein histone macroH2A (mH2A) or formation of DNA damage foci containing activated histone H2A.X (g-H2A.X) have long been used as markers of cellular senescence. An age-dependent increase in mH2A has been detected in several organs/tissues in mice and baboons, including lung, liver, gut epithelium, and skeletal muscle [29,30]. It has also been reported that the expression of mH2A is significantly increased with age in the lung in mice and humans, which is accompanied by a significant increase in the deposition of matrix proteins, as well as lung dynamic compliance and airway resistance [31]. Treatment of mice with rapamycin blocked the mTOR activity, prevented cellular senescence, and reduced lung collagen deposition [31], suggesting that cellular senescence may contribute to the aging-related decline in lung function.

Stem cells can proliferate and differentiate into different types of cells upon stimulation under either physiological or pathological conditions. Stem cell proliferation is essential for tissue repairing, regeneration, and maintenance. One of the hypothetic mechanisms by which cellular senescence contributes to aging, therefore, is that stem cells undergo senescence and lose regeneration capacity. Studies have shown that the expression of p16 increases with age in hematopoietic stem cells (HSCs) and brain progenitor cells [32,33]. This is associated with decreased cell repopulating activity and increased apoptosis upon stress in HSCs [32], as well as a decreased proliferation of multiple progenitor cells in the mouse brain [33]. Ablation of the p16^INK4a^ gene, on the other hand, mitigated aging-associated HSC dysfunction and improved survival of the animal in successive transplants, a stem-cell-autonomous tissue regeneration model [32]. p16^INK4a^ deficient mice also show mitigated the aging-related decline in self-renewal potential of multipotent brain progenitors [33]. Together, these results suggest that the age-dependent increase in p16 positive (senescent) stem and progenitor cells may contribute to the age-related decline in brain and bone marrow functions.

To better understand the role of cell senescence in aging and aging-related diseases, a transgenic mouse model INK–ATTC that expresses FK506-binding protein Caspase 8 fusion protein under the control of p16^Ink4a^ promotor has been generated [8]. Using this novel mouse model, Backer et al. showed, for the first time, that removal of naturally occurring p16^Ink4a^ positive cells by administration of AP20187 delayed the onset of aging-related pathologies in various tissues/organs, including adipose tissue, skeletal muscle, and eye [8]. They also showed that late-life clearance of p16^INK4a^ positive cells slowed down the progression of already established aging-related disorders [8]. Their data suggest that cellular senescence is causally implicated in aging-related phenotypes and that removal of senescent cells can prevent or delay aging-related organ/tissue dysfunction [8]. In another study, the same group further showed that removal of p16^Ink4a^-expressing cells by injection of AP20187 to INK–ATTAC transgenic mice extended the median lifespan of both male and female mice with two distinct genetic backgrounds [13]. Clearance of p16^Ink4a^ positive cells also attenuated the age-related deterioration of several organs including the kidney, heart, and fat [13]. These data further suggest that p16^Ink4a^ positive cells that accumulate during adulthood negatively influence lifespan and promote aging-related pathologies in different organs.

In addition to genetic approaches, pharmacological approaches have also been used to remove senescent cells to elaborate the role of cell senescence in aging. Xu et al. reported that transplanting a small number of senescent cells into young mice caused persistent physical dysfunction and induced host tissues to undergo senescence [14]. They further showed that fewer senescent cells were required to cause the same effects and to reduce survival in old mice, suggesting that senescent cells can pass their signals to adjacent cells and that cellular senescence is related to the shortening of the lifespan [14]. Importantly, the authors showed that treatment of senescent-cell transplanted young mice or naturally aged mice with senolytic drugs dasatinib and quercetin, which selectively eliminate senescent cells through inducing apoptosis of senescent cells, alleviated physical dysfunction and increased post-treatment survival [14]. Their data provide proof-of-concept evidence that cell senescence is associated with physical dysfunction and reduction in survival in old age. Using genetic and pharmacological approaches, Ogrodnik et al. also showed that removal of senescent cells improved cognitive function in aged mice [19]. Together, these results suggest that cellular senescence contributes importantly to the aging-related decline in organ function as well as lifespan. Removal of senescent cells may promote healthy aging and extend life.

## 3. Cellular Senescence and Alzheimer’s Disease

Alzheimer’s disease (AD) is an aging-related neurodegenerative disease and a major cause of dementia in the elderly [34]. Over 30 million people worldwide suffer from AD, and there is no effective treatment for AD due to an incomplete understanding of its etiology and pathogenesis [35,36]. It is estimated that the incidence of AD doubles every 5 years after age 65, and 50% of the population aged 85 or older suffer from AD. Therefore, aging is considered the greatest risk factor for AD, although the mechanism underlying the aging-related susceptibility to AD is unknown. Evidence from both human and animal studies indicates that cellular senescence plays a critical role in the development of many aging-related diseases [7,8,9,10,11,12,13,14,15], including AD [18,24,25,36,37,38,39,40,41,42,43]. Senile plaques, which are extracellular deposits of β-amyloid (Aβ) peptides, and neurofibrillary tangles (NFTs), which are intracellular accumulation/deposition of hyperphosphorylated tau proteins, are two neuropathological features of AD [44,45]. Although it is still debatable whether and how Aβ and hyperphosphorylated tau lead to neurodegeneration, a foundation of memory loss in AD, accumulating evidence indicates that both Aβ and tau pathologies are potent inducers of cellular senescence. Senescent cells have been detected in the brain of AD patients [18,25,36,37,38,39,41,42,43] and AD model mice that overexpress Aβ or tau protein [24,25,40,41]. Removal of senescent cells pharmacologically and genetically reduced brain Aβ load and tauopathy and improved memory in these AD model mice [24,25,41]. These data strongly suggest that cell senescence mediates Aβ- and tauopathy-induced neuropathophysiology in AD. These data also suggest that cell senescence promotes Aβ and tau pathologies. Elucidation of the mechanisms underlying brain cell senescence during aging and in AD, as well as the mechanism by which senescent cells contribute to neurodegeneration in AD, will be key to the development of strategies for the prevention and treatment of this devastating disease.

### 3.1. Evidence of Cellular Senescence in AD Patients

Although there is increasing evidence showing brain cell senescence in the animal models of AD, the evidence of cell senescence in the brain of AD patients is still scanty. This is, in part, due to lack of specific and sensitive markers for senescent cells in vivo and in postmortem samples. Nonetheless, several studies, mostly using immunostaining techniques, have shown that different types of cells, including astrocytes, microglia, neurons, and endothelial cells, in the AD brain express high levels of senescence-associated proteins, including cell cycle repressors p16, p53, and p21 [18,25,38,39,41,42,46,47,48,49,50], suggesting that cellular senescence may play a role in AD pathophysiology.

Naderi et al. reported that fibroblasts isolated from AD patients express higher levels of p21 but decreased amount of Bax, an apoptosis marker, compared with fibroblasts isolated from non-AD individuals [46]. Senescent endothelial cells have also been detected in the frontal and temporal cortex of AD patients [48]. Turnquist and Bhat showed that the numbers of p53β, a coactivator of full-length p53, and p16 positive astrocytes were increased in the brain of AD patients and these senescent astrocytes secreted a number of cytokines, including IL-6, a senescence marker [18,47]. Zhang et al. reported, on the other hand, that oligodendrocyte progenitor cells, not astrocytes, microglia, or oligodendrocytes, in the AD brain exhibited senescence-like phenotype, characterized by increased expression of p21 and p16 and increased activity of SA-β-gal [25]. The sustained proliferation of microglia is a hallmark of AD. A recent study demonstrated that early and sustained proliferation promoted replicative senescence phenotypes in microglia, characterized by increased SA-β-gal activity, telomere shortening, and senescence-associated transcriptional signature, in APP/PS1 mice, a well-established murine model of familial AD, and in postmortem tissue of AD patients [50]. Suppression of early microglial proliferation hindered the development of microglial senescence, Aβ accumulation, neuritic and synaptic damage in APP/PS1 mice [50]. Their data suggest that sustained proliferation may trigger replicative senescence of microglia in the AD brain.

Hyperphosphorylation of Tau proteins, which leads to the formation/deposition of neurofibrillary tangles (NFTs) inside cells, is one of the pathological hallmarks of the AD brain. Using laser capture microdissection techniques, Musi et al. showed that neurofibrillary tangle (NFT)-bearing neurons, not neurons surrounding Aβ plaques, displayed senescence-like phenotype in tau transgenic mice and AD brain tissue [41]. Gaikwad et al. also showed recently that astrocytes surrounding pathological tau oligomers displayed senescent phenotype in the AD brain [49]. Vascular dysfunction, mainly endothelial dysfunction, plays a critical role in the pathogenesis and progression of AD. Bryant et al. compared the expression of 42 genes that are associated with cellular senescence and adhesion in the intact microvessels isolated from the dorsolateral prefrontal cortex of 16 subjects with advanced AD (Braak V/VI, B3) and 12 healthy controls. They found that several proteins associated with cellular senescence and adhesion, including Serpine1 (also called PAI-1), a serine protease inhibitor and cell senescence marker [51,52,53,54,55,56,57], were significantly increased in B3 microvessels from AD patients, compared with that from the controls after adjusting for sex and cerebrovascular pathology [58]. Their data suggest that endothelial cells may undergo senescence in AD patients.

A telomere is a region of tandem repeats of short DNA sequences at the end of a chromosome, which protects the chromosome from erosion. During the cell cycle, an incomplete DNA replication leads to the loss of a part of the telomere and thereby instability of the chromosome. Telomere shortening leads to a permanent cell cycle arrest, which is known as replicative senescence. Numerous studies have shown that telomere shortening is associated with aging and aging-related diseases, including AD [59,60,61,62,63,64,65], although no-association results have also been reported [66,67]. Lee et al. reported that peripheral leukocyte DNA from individuals with AD has a greater telomere shortening rate per year than leukocytes from healthy individuals and individuals with mild cognitive impairment (MCI) [64]. Liu et al. showed that the negative correlation between telomere length and age was much stronger in AD patients, especially in females, than in the control group [68]. Interestingly, it has also been shown that telomere length is significantly shorter in AD patients bearing homozygous *ApoEɛ4* allele than AD patients bearing heterozygote *ApoEɛ4*, although no significant difference in telomere length is detected between AD patients and nondementia elderly control in general [69]. Using the genetic risk score and Mendelian Randomization (MR) techniques, Guo et al. showed that shorter telomere length was causally associated with a higher risk of AD [61]. Telomere shortening has also been shown to be associated with a rapid cognitive decline and conversion to dementia in MCI patients [65]. Nonetheless, no-association results have also been reported. Moverare-Skrtic et al. reported that leukocyte telomere length (LTL) was reduced in stable MCI, but LTL shortening was not associated with conversion to AD [66]. In a longitudinal community-based age-cohort study, Hinterberger et al. reported that leukocyte telomere length was linked to vascular diseases but not AD [67].

### 3.2. Evidence of Cellular Senescence in AD Model Mice

Increased deposition of amyloid plaques extracellularly and accumulation of hyperphosphorylated tau proteins intracellularly are two pathological hallmarks of Alzheimer’s disease [44,45]. Although the mechanisms underlying increased deposition of Aβ and phosphorylated tau proteins in the brain of LOAD patients are unknown, several mouse models that overexpress mutant human amyloid precursor protein (APP) or APP plus mutant human presenilin 1 or mutant form of human microtubule-associated protein tau P301S have been generated and used to study the mechanisms, whereby Aβ and hyperphosphorylated tau proteins promote AD pathophysiology [24,25,40,41,49,70,71,72,73,74,75,76]. Emerging evidence from these animal studies indicates that increased Aβ and tau phosphorylation promote brain cell senescence and that cellular senescence plays a critical role in the neuropathology and memory decline in AD.

### 3.3. Mice Overexpressing Amyloid Precursor Protein (APP)

Several mice or rat models have been generated to overexpress human APP or APP plus mutant human presenilin 1 to study the role of Aβ accumulation in AD neuropathophysiology. APP/PS1 mice are double transgenic mice expressing a chimeric mouse/human APP (Mo/HuAPP695swe) and a mutant human presenilin 1 (PS1-dE9). These mice display a significant increase in brain Aβ load and memory impairment and have been widely used as a murine model of familial AD. Emerging evidence shows that various types of brain cells, including oligodendrocyte precursor cells (OPCs), astrocytes, microglia, and neurons, express higher levels of senescence markers p16, p21, and SA-β-gal in these familial AD model mice [25,40,70,71] Nicotinamide adenine dinucleotide (NAD+) is an important metabolite and is involved in many biological processes. Hou et al. reported that, associated with a decline in NAD+ level in the brain, various types of brain cells in APP/PS1 mice displayed a senescence phenotype, including increased SA-β-gal activity, as well as increased p16 and p21 expression, [71]. Treatment with NAD+ precursor nicotinamide riboside (NR) increased brain NAD+ level and reduced senescent cell number and neuroinflammation, suggesting that cellular senescence and neuropathological changes were observed in the brain of APP/PS1 mice may result from NAD+ deficiency [71]. A causative relation between cellular senescence and neuropathophysiology in APP/PS1 mice has been well elaborated in another recent publication [25]. Zhang et al. demonstrated that Aβ-plaque-associated Olig2- and NG2-expressing OPCs, but not astrocyte, microglia, or oligodendrocytes, in the brains of AD patients and APP/PS1 mice exhibit a senescence-like phenotype, characterized by increased SA-β-gal activity and increased expression of two cell cycle repressors p16 and p21 [25]. Direct exposure of cultured OPCs to aggregated Aβ induced senescence in these cells [25]. Most importantly, they showed that treatment of APP/PS1 mice with senolytic drugs selectively removed senescent cells from the plaque environment, reduced neuroinflammation and Aβ load, and ameliorated memory deficits [25]. Their data strongly suggest that cell senescence plays a pivotal role in Aβ-mediated neuropathology and memory deficit. Their results also suggest that cell senescence contributes to Aβ accumulation in the AD brain.

### 3.4. Tau Transgenic Mice

Several mouse models expressing wild-type or mutant human tau protein have been used to study the mechanism by which tauopathy contributes to the neuropathophysiology of AD [24,41,49,73,74,75,76]. One of these mouse models is MAPT^P301S^PS19 (PS19) mice, which express the P301S mutant form of the human microtubule-associated protein tau (*MAPT*) under the direction of the mouse prion protein promoter. These mice express high levels of mutant human tau specifically in neurons [75] and display gliosis, neurofibrillary tangle (NFT) deposition, neurodegeneration, and loss of cognitive function [24]. Bussian et al. reported that astrocytes and microglia in the brain of PS19 mice expressed high levels of senescence marker p16 [24]. Most importantly, they showed that clearance of p16 expressing senescent cells genetically by crossing PS19 mice with INK–ATTAC transgenic mice or pharmacologically by administration of senolytic drugs prevented gliosis, tau hyperphosphorylation, and degeneration of cortical and hippocampal neurons, associated with preservation of memory function [24]. These results provide a clear causal link between tauopathies, cellular senescence, neurodegeneration, and memory loss.

rTg4510 mice express mutant human tau^P301L^ in the forebrain. These mice develop tau pathology in forebrain regions, concomitant with neurodegeneration and cognitive deficits [74]. Musi et al. showed that NFT-bearing, but not Aβ-plaque-associated, neurons displayed a senescence-like phenotype and expressed senescence-related genes, including Cdkn2a, which was directly correlated with brain atrophy and NFT burden [41]. Most importantly, they showed that treatment of these mice with senolytic drugs dasatinib and quercetin reduced total NFT density, neuron loss, and ventricular enlargement, suggesting that cellular senescence is involved in tauopathy-induced neuron loss [41].

In Alzheimer’s disease, the accumulation of neurofibrillary tangles occurs in the absence of tau mutations. To study the pathogenesis of overexpression of normal tau protein, hTau mice, which express six isoforms of human MAPT protein but not mouse tau, have been generated [73]. These mice develop age-dependent AD-like pathology, including accumulation of hyperphosphorylated tau as aggregated paired helical filaments in cell bodies and dendrites of neurons, neuroinflammation, and synapse dysfunction, as well as memory deficit [49,73]. Importantly, these mice have increased loads of senescent cells in the brain [49]. Treatment of hTau mice with inhibitors to high mobility group box 1 (HMGB1), which mediated tau-protein-induced astrocyte senescence in vitro, significantly decreased senescent cell load in the brain, reduced neuroinflammation, and ameliorated cognitive dysfunctions [49]. These data further support the notion that tau protein promotes memory loss at least in part by inducing brain cell senescence.

## 4. Potential Mechanisms Underlying Brain Cellular Senescence in AD

**Figure 2:**
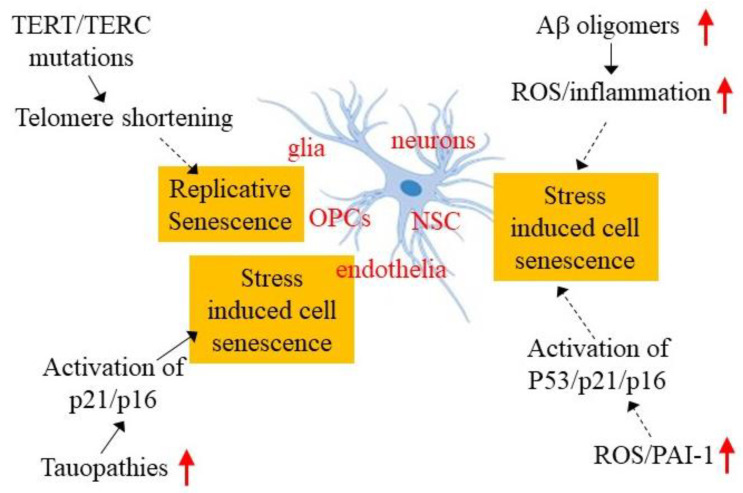
Potential mechanisms underlying brain cell senescence in AD. Various types of cells, including glia, oligodendrocyte precursor cells (OPCs), neuronal stem cells (NSCs), neurons, and endothelia, have been shown to undergo senescence in AD brain and/or AD model mice. Mutations in the telomerase reverse transcriptase (TERT) gene and telomerase RNA component (TERC), which leads to telomere shortening, hyperphosphorylated tau, increased Aβ oligomers, reactive oxygen species (ROS), and plasminogen activator inhibitor 1 (PAI-1) expression, are implicated in brain cell senescence in AD. Red arrows indicate increases in the amounts of these molecules.

The mechanisms underlying cell senescence in either physiological or pathological conditions remain unclear. Although telomere shortening is believed to be responsible for replicative senescence in proliferating cells, the molecular mechanism linking telomere shortening to cell senescence is still not well defined. Furthermore, although it has been well documented that various stress conditions, including DNA damage, reactive oxygen species (ROS), mitochondrial dysfunction, and lysosomal alteration, induce premature cell senescence, the signaling pathways leading to cell senescence under these different stress conditions remain poorly understood. In this section, we summarize recent findings regarding the potential mechanisms underlying brain cell senescence in AD (Figure 2).

### 4.1. Telomerase Deficiency and Telomere Shortening

Shortening of telomeres at the ends of mammalian chromosomes because of cellular replication leads to a permanent cell cycle arrest, which is also known as replicative senescence. Numerous studies have shown that telomere lengths are shorter in AD patients, compared with age-matched health controls [59,60,61,62,63,64,65], suggesting a potential role of telomere shortening in cell senescence in AD. The mechanism underlying telomere shortening in AD is unknown. Mata al. reported that leukocyte telomere length (LTL) is reduced in MCI patients and reduced even more in AD patients, compared with healthy controls [62]. They also found that serum folate concentration was positively associated with LTL, whereas serum homocysteine level was negatively associated with LTL, suggesting that shortened LTL in MCI and AD may be related to the changes in the serum folate and homocysteine levels [62]. Microglia are self-renewable. A recent study demonstrated that early and sustained proliferated microglia displayed shortened telomeres in APP/PS1 mice, indicating replicative senescence of microglia in these AD model mice [50]. These data suggest that microglia in the AD brain may undergo replicative senescence due to sustained proliferation and thus accelerated telomere shortening.

Telomerase, consisting of telomerase reverse transcriptase (TERT) and telomerase RNA component (TERC), restores the telomere length and, therefore, plays a critical role in DNA stability and cell proliferation. It has been reported that the polymorphisms of the TERT gene are associated with increased risk of AD in man, while three TERC gene polymorphisms are in strict linkage disequilibrium, and their genotype combinations influence the age of AD onset [77]. Using a telomerase deficient mouse model, Whittemore et al. studied telomerase deficiency and aging phenotypes [78]. They found that old telomerase deficient mice exhibited the signs of neurodegeneration; delivery of the telomerase gene to their brain, on the other hand, ameliorated some of the neurodegeneration phenotypes [78]. Together, the results suggest that telomere shortening in AD, at least in part, results from mutations of telomerase genes. The molecular mechanism by which telomere shortening induces cell senescence remains unclear. Herbig et al. showed that telomeric foci in fibroblasts contain multiple DNA damage response factors, including ataxia telangiectasia mutated (ATM) and p53, which were assembled in a subset of senescent cells [79]. Inhibition of ATM expression or activity resulted in cell cycle reentry, suggesting that telomere shortening may induce cell senescence through activating the ATM–p53 cell senescence pathway [79].

### 4.2. Aβ Oligomers

Accumulation of β-amyloid (Aβ) peptides in the brain is a pathological feature of AD [44,45]. Although the mechanism by which Aβ promotes neuronal degeneration and memory loss in AD is still controversial, accumulated evidence indicates that Aβ is a potent inducer of cell senescence [25,40,50,70,71,80,81,82,83,84,85,86]. Wei et al. showed that the expression of cell senescence marker p16 is significantly increased in neurons, although not in astrocytes or microglia, in 5XFAD transgenic mice, which overexpress human amyloid β (A4) precursor protein 695 (APP) with three mutations detected in familial Alzheimer’s disease (FAD) and human presenilin 1 (PS1) harboring two FAD mutations [40]. They also showed that treatment with oligomeric Aβ induces p16 in cultured primary neurons, consistent with in vivo mouse data [40]. These data indicate that Aβ oligomers can directly induce neuron senescence. APP/PS1 mice, which overexpress mutant human APP and PS1 genes and develop brain amyloidosis at early life, is another widely used FAD mouse model. Several studies have shown that the numbers of senescent cells are increased in the brain of APP/PS1 mice [25,50,70,71], strongly suggesting that increased Aβ can provoke cellular senescence in vivo.

In addition to these Aβ overproducing mice, numerous in vitro studies have also shown that Aβ oligomers induce neuron or endothelial cell senescence in culture [81,82,83,84,85,86]. Singh Angom reported that Aβ1-42 oligomers, not monomer or fibrils, induced brain endothelial cell senescence through upregulation of vascular endothelial growth factor 1 (VEGF-1) [84]. Similarly, He et al. reported that treatment with Aβ1-42 oligomers induced p16 and SA-β-gal in adult mouse hippocampal neural stem/progenitor cells (NSPCs) [81]. Blocking Aβ receptor with WRW4 abrogated Aβ1-42 oligomers-induced NSPC senescence, suggesting that Aβ42 induces NSPC senescence through binding to this Aβ receptor [81]. Other investigators have shown that Aβ promoted neural cell senescence by increasing reactive oxygen species (ROS) production [83,85]. Neuroinflammation plays a critical role in AD pathology. Shang et al. reported that treatment of rat astrocytes with Aβ-increased IL-1β, an important neuroinflammatory cytokine that is increased in AD brain, and cell senescence [86]. Their studies further suggest that Aβ promotes astrocyte senescence probably through activating NLR family pyrin domain containing 3 (NLRP3) pathway, thus increasing IL-1β production [86].

### 4.3. Tauopathy

Although the mechanism by which hyperphosphorylated tau proteins promote neurodegeneration in AD is still under debate, emerging evidence suggests that inducing cellular senescence may be a key intermediate [87]. Tau pathology has been shown to be associated with senescence of various types of cells, including astrocytes, microglia, OPCs, and neurons in the brains of AD patients and AD animal models [24,41,47,49,87]. Neurons are terminally differentiated cells. p16 is normally not expressed in terminally differentiated neurons. However, studies have shown that neurons that contain neurofibrillary tangles in the brain of AD patients express increased levels of CDK4 and p16 [37,88], suggesting that re-entry of cell cycles makes these cells sensitive to tauopathy-induced cell senescence. Musi et al. have also reported similar results that tau-containing NFTs trigger a cascade of events that are highly correlated to cell senescence in NFT containing neurons in human and mouse brains [41]. Although tau pathology is observed mostly inside neurons, some studies have shown that tau pathology is also associated with glia cell senescence in the brain of AD patients [47,49]. Tau oligomers-containing astrocytes with senescent phenotypes have been demonstrated in the brain of AD patients [49]. Streit et al. further showed that senescent, not activated, microglial cells were associated with tau-positive, degenerating neurons in the AD brain [47], suggesting that tauopathy-induced pathological changes in neurons may further provoke senescence in surrounding astrocytes. Although the mechanism by which Tau protein promotes cell senescence is unclear, it has been reported that tau-bearing NFT triggers neuron senescence probably through activation of Cdkn1a (p21) and Cdkn2a (p16), two cell cycle inhibitors. It is worth mentioning that a recent study showed that genetic or pharmacological removal of senescent cells alleviated gliosis and cognitive deficit and also diminished NFT in a well-characterized tauopathy mouse model, PS19 mice, suggesting that cell senescence, in turn, facilitates tauopathy, forming a vicious cycle [24].

### 4.4. Oxidative Stress

Oxidative stress, an imbalance between oxidant production and antioxidant defense, increases with age and is believed to contribute importantly to aging and aging-related diseases, including AD [89,90,91,92,93]. Reactive oxygen species (ROS) have been shown to induce senescence in different types of cells, although the underlying mechanism is not well understood. Numerous studies have shown that ROS can induce or mediate Aβ-induced brain cell senescence in AD model mice and in cultured brain cells [81,83,85,94,95]. He et al. reported that treatment with Aβ1-42 oligomers induced senescence in adult mouse hippocampal neural stem/progenitor cells (NSPCs) [81]. Blocking Aβ receptor with WRW4 abrogated Aβ1-42 oligomers-induced ROS production, p38 map kinase activation, and NSPC senescence, suggesting that Aβ42 induces NSPC senescence probably through increasing ROS and activating p38 pathway [81]. Zhang et al. reported that the Aβ1-40 treatment increased ROS production and induced p53 in U87 cells, a human astrocyte cell line derived from malignant gliomas [83]. Treatment with galantamine alleviated Aβ1-40-induced ROS production, p53 expression, and U87 cell senescence, suggesting that ROS mediates Aβ-induced U87 cell senescence [83]. Studies from other groups showed that Aβ oligomers induced neuron senescence also through increasing ROS production [85,95]. Li et al. reported that Aβ42 oligomers increased ROS production, mitochondrial protein acetylation, and senescence in a neuroblastoma cell line SK–N–SH cells [85]. Treatment with a newly synthesized rhamnoside derivative PL171 inhibited Aβ42 oligomers-induced ROS production, acetylation of mitochondrial proteins, and neuronal cell senescence [85]. They also showed that PL171 exerted its protective effects through upregulation of Sirt3 as inhibition of Sirt3 activity eliminated the protective effect of PL171 [85]. Similar results have been reported with M17 neuronal cells [95].

### 4.5. Increased Expression of Plasminogen Activator Inhibitor 1 and Cell Senescence

Plasminogen activator inhibitor 1 (PAI-1, also called Serpine 1) is a physiological inhibitor of tissue-type and urokinase-type plasminogen activators (tPA and uPA), which convert plasminogen into plasmin, a serine protease playing a central role in hemostasis. Previous studies from this lab and others have shown that PAI-1 protein levels are increased in the plasma of the elderly and in the AD brain [15,96,97,98,99,100,101,102,103,104]. Animal studies, including ours, further showed that PAI-1 is increased with age in wild-type animals and familial AD model mice [15,24,100,105,106,107,108]. Inhibition of PAI-1 activity or deletion of the PAI-1 gene reduced brain Aβ load and improved memory in APP/PS1 mice [15,100,107,109], suggesting a pivotal role of PAI-1 in the pathophysiology of AD, although the mechanism by which PAI-1 promotes AD-liked neuropathophysiology remains unclear. Notably, PAI-1 expression is increased in many senescent cells, and increased PAI-1 has long been used as a marker of cell senescence in vitro and in vivo [13,24,110,111,112,113]. Most importantly, accumulated evidence indicates that increased PAI-1 is not merely a marker but also a mediator of cell senescence [51,52,53,54,55,56,57]. Our previous data showed that increased PAI-1 expression/activity mediated alveolar type II (ATII) cell senescence through activating p53-p21–pRb cell cycle repression pathway in bleomycin-induced lung fibrosis model [55]. In another study, we further showed that PAI-1 was involved in transforming growth factor β1 (TGF-β1)-induced ATII cell senescence through inducing p16, not p53 [57]. Importantly, several recent studies have shown that PAI-1 expression is increased in senescent cells in the brain of AD patients [50,58] and AD model mice [24], suggesting that increased PAI-1 contributes to neuropathophysiology of AD probably through inducing brain cell senescence. Further studies are required to prove this hypothesis.

## 5. Limitation and Future Directions

Emerging evidence from human samples and animal studies indicates that cellular senescence contributes importantly to aging and aging-related diseases, including AD. The mechanisms underlying brain cell senescence and by which cell senescence contributes to neurodegeneration and memory loss in AD, however, remain largely unknown. One of the challenges faced in the field is the detection of senescent cells in vivo and in postmortem samples. Most of the cell senescence data from postmortem samples were obtained by immunohistochemistry staining, which is not specific or sensitive. More sensitive and specific methods are to be developed to identify senescent cells in vivo. Defining the cell types that undergo senescence in the AD brain will provide mechanistic insights into the pathophysiology of the disease. Single-cell or single nuclear RNA-sequencing techniques have been well established in recent years; however, no single cell or single nuclear RNA-sequencing data about cell senescence in aging or AD brain has been reported yet. Finally, several studies have shown promising results with senolytic drugs. Whether these drugs can be used in clinics to extend the lifespan and/or ameliorate aging-related pathophysiological changes remains to be determined.

## Data Availability

No need as this is a review article.

## Abbreviations

LOAD	Late-onset Alzheimer’s disease
AD	Alzheimer’s disease
Aβ	β-amyloid peptides
PAI-1	Plasminogen activator inhibitor 1
RS	Replicative senescence
SIPS	Stress-induced premature senescence
SASP	Senescence-associated secretory phenotype
SA-β-gal	Senescence-associated β-galactosidase
pRb	Phosphorylated retinoblastoma protein
SAHFs	Senescence-associated heterochromatin foci
SDFs	Senescence-associated DNA damage foci
mH2A	MacroH2A
HSCs	Hematopoietic stem cells
NFTs	Neurofibrillary tangles
MCI	Mild cognitive impairment
LTL	Leukocyte telomere length
APP	Amyloid precursor protein
PS1	Presenilin 1
OPCs	Oligodendrocyte precursor cells
HMGB1	High-mobility group box 1
TERT	Telomerase reverse transcriptase
TERC	Telomerase RNA component
ATM	Ataxia-telangiectasia mutated
FAD	Familial AD
VEGF-1	Vascular endothelial growth factor 1
NSPCs	Neural stem/progenitor cells
ROS	Reactive oxygen species
NLRP3	NLR family pyrin domain containing 3
DAM	Disease-associated microglia
tPA	Tissue-type plasminogen activator
uPA	Urokinase-type plasminogen activator inhibitor
ATII	Alveolar type II cells
TGF-β1	Transforming growth factor β1
NSC	Neuronal stem cell
